# Face Masks and Respirators in the Fight Against the COVID-19 Pandemic: A Review of Current Materials, Advances and Future Perspectives

**DOI:** 10.3390/ma13153363

**Published:** 2020-07-29

**Authors:** Kris O’Dowd, Keerthi M. Nair, Parnia Forouzandeh, Snehamol Mathew, Jamie Grant, Ruth Moran, John Bartlett, Jerry Bird, Suresh C. Pillai

**Affiliations:** 1 Nanotechnology and Bio-Engineering Research Group, Department of Environmental Science, Institute of Technology Sligo, F91 YW50 Sligo, Ireland; kris.odowd@mail.itsligo.ie (K.O.); keerthi.nair@mail.itsligo.ie (K.M.N.); parnia.forouzandeh@mail.itsligo.ie (P.F.); Snehamol.Mathew@mail.itsligo.ie (S.M.); James.Grant2@mail.itsligo.ie (J.G.); moran.ruth@itsligo.ie (R.M.); 2 Faculty of Science, Institute of Technology Sligo, F91 YW50 Sligo, Ireland; bartlett.john@itsligo.ie (J.B.); bird.jerry@itsligo.ie (J.B.)

**Keywords:** SARS-CoV-2, facemasks, droplets, respirators, legislations, testing, reuse, modelling, personal protective equipment (PPE)

## Abstract

The outbreak of COVID-19 has spread rapidly across the globe, greatly affecting how humans as a whole interact, work and go about their daily life. One of the key pieces of personal protective equipment (PPE) that is being utilised to return to the norm is the face mask or respirator. In this review we aim to examine face masks and respirators, looking at the current materials in use and possible future innovations that will enhance their protection against SARS-CoV-2. Previous studies concluded that cotton, natural silk and chiffon could provide above 50% efficiency. In addition, it was found that cotton quilt with a highly tangled fibrous nature provides efficient filtration in the small particle size range. Novel designs by employing various filter materials such as nanofibres, silver nanoparticles, and nano-webs on the filter surfaces to induce antimicrobial properties are also discussed in detail. Modification of N95/N99 masks to provide additional filtration of air and to deactivate the pathogens using various technologies such as low- temperature plasma is reviewed. Legislative guidelines for selecting and wearing facial protection are also discussed. The feasibility of reusing these masks will be examined as well as a discussion on the modelling of mask use and the impact wearing them can have. The use of Artificial Intelligence (AI) models and its applications to minimise or prevent the spread of the virus using face masks and respirators is also addressed. It is concluded that a significant amount of research is required for the development of highly efficient, reusable, anti-viral and thermally regulated face masks and respirators.

## 1. Introduction

### 1.1. COVID-19 Pandemic

The COVID-19 pandemic broke out in December of 2019 in a city called Wuhan in the Hubei province of China [1]. COVID-19 is a pneumonia-based disease that is the result of the infection of severe acute respiratory syndrome coronavirus 2 (SARS-CoV-2). This is believed to be related to Middle East respiratory disease (MERS) and severe acute respiratory syndrome (SARS) [2]. The origin of the disease is suspected to be from a seafood market, however this has not been corroborated [3]. The pandemic was declared a global health emergency by the World Health Organisation (WHO) on the 30th of January [4] with the coronavirus obtaining its official name of COVID-19 on the 11th of February [5] by the WHO’s director general. As of the 17th of July 2020, there have been 13,788,300 reported cases worldwide with 589,688 deaths [6]. On the 19th of March, WHO issued guidelines for the prevention of infection of COVID-19. They recommended that health care workers use medical masks when treating suspected patients and that these patients also wear masks or covered their mouths when coughing or sneezing [7]. The main routes of infection are believed to be from “respiratory fluid droplets” [8] containing the virus that are between 10 and 5 µm and through aerosols that are less than 5 µm [9]. These droplets can spread the viruses through three different methods; airborne, contact and fomites. Airborne transmission results in direct infection due to the inhalation of droplets in the air. Contact transmission is a result of the droplets landing on an individual and then being transmitted to an area where infection can occur, for example, a hand touching a face. Both airborne and contact droplets can be transmissible within 1 m of an individual who is infected with the virus and coughs or sneezes [10]. Fomites are an indirect method whereby the droplets land on a surface and are then transmitted to an individual, for example a door handle [8]. In previous influenza infections, the effectiveness of each transmission method was not ranked and guidelines have informed healthcare workers to be vigilant against all forms of transmission by wearing N-95 respirators [11].

**Figure 1:**
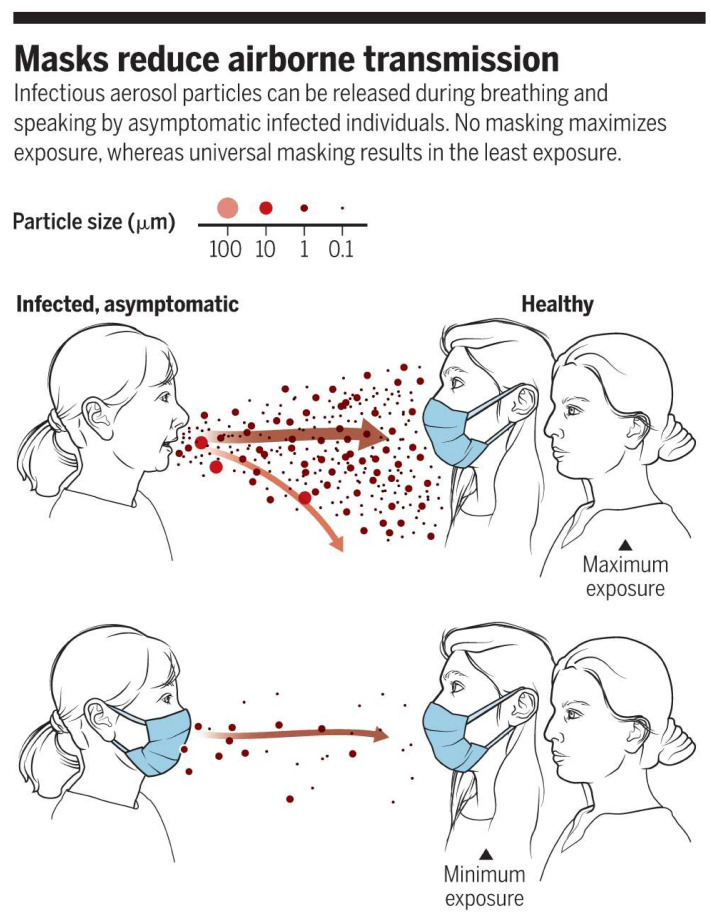
How face masks help reduced airborne transmission Reproduced with permission from reference. [9]. Copyright (2020), Science.

Respiratory droplets and aerosols (Figure 1) are the main method of infection of COVID-19 with asymptomatic individuals capable of transmitting the virus unknowingly whilst breathing or speaking [9]. The size of these droplets alters the method of infection, large droplets (>20 µm) will fall onto objects easier than smaller droplets due to gravity while small droplets (<5–10 µm) will evaporate midair allowing for airborne transmission [12]. Droplets of 1 µm in size were reported to be capable of staying airborne for more than 12 h with strong coughs or sneezes capable of sending these particles over 20 feet [9]. A study found that 40% and 30% of aerosols and respiratory droplets from individuals with coronavirus tested positive for coronavirus respectively when the subjects were not using a mask. When the individual wore a face mask, however, they were unable to detect any coronavirus in either the aerosol or the droplets [13]. This would indicate that the use of a face mask can help prevent the spread of the virus from infected individuals.

In Ireland, the current stance on the use of face masks is that it is recommended but not an enforceable law [14]. The department of health advises the use of a cloth face covering when people are unable to stay two meters apart and when they are in an enclosed space. They have issued designs for the general public so as to create face masks at home to be used [15]. The use of respirators is only recommended for health care workers to maintain a sufficient supply. Within the health care sector, face masks are to be used by staff in pathology labs and when near patients with respiratory symptoms. Respirators are to be used when there is a risk of an aerosol generating procedure [16]. The compulsory use of face masks has been implemented in several European countries, with different countries enforcing different levels. Some countries have made wearing masks mandatory on public transport and shops, such as the Czech Republic [17] and Germany [18], while countries like Spain [19] have made them mandatory to be worn outside as well. In the United States of America, the Centre for Disease Control and Prevention have recommended that face masks be worn when social distancing cannot be upheld and for respirators and surgical masks not to be used [20].

### 1.2. Face Masks

The first recorded use of medical masks comes from a Polish surgeon Jan Mikulicz Radecki in 1897 with his assistant publishing the following year how the masks could reduce the spread of droplets from the user’s mouth [21]. This mask was comprised of a single layer of gauze to cover the mouth [22]. In 1899, Flüge developed masks where the mouth was covered by strips of roller gauze [23]. It was noted in 1898 that increased layers of gauze increased the protective quality of the mask [22]. The use of a “mouth guard” was developed in 1905 to prevent the transfer of tuberculosis by stopping droplets of sputum [24]. By the 1930s, specific requirements were created for masks; 1) to be low cost and washable, 2) to be comfortable and cover the mouth and nose, and not to cause fogging of glasses and 3) they should not allow the transfer of organisms across the material. During this time, deflector style masks began development using different styles of material placed between gauze to enhance the mask [22]. With further developments of masks with a filter and paper-based masks, new filter-based mask proved to be the more effective.

Disposable masks were developed in the 1960s made as a moulded shell [25] followed by masks containing polypropylene or fibre glass filters. This change to disposable masks became cemented in the 1970s when 75% of all masks being used were disposable. Today the use of a muslin or gauze mask is rarely seen.

### 1.3. Respirators

**Figure 2:**
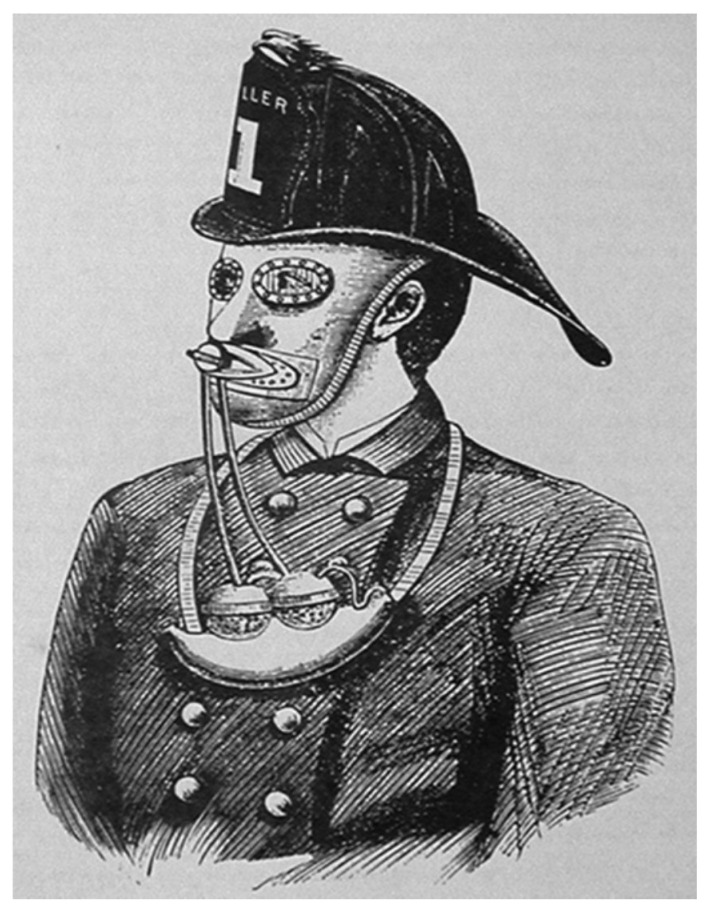
“Nearly” smoke mask created in 1877. Reproduced with permission from reference [26]. Copyright (2012), Elsevier.

Respirators have been in use since the time of the Romans whereby a wet cloth was used in lead mines to prevent inhalation of toxic chemicals [26]. In 1877, one of the first respirators (Figure 2) was developed for fires called the “Nearly” smoke mask [26].

Their use in medical based work did not come about until the 1980s when Mycobacterium tuberculosis (TB) infections became prevalent in the United States of America [27]. Respirators were used instead of traditional face masks in TB quarantined zones and became a part of the Occupational Safety and Health Administration (OSHA) guidelines for dealing with TB in 1997. The use of “Surgical N95 respirators” obtained approval in 2002 by the FDA followed by their use to help decrease infection of any airborne based diseases.

## 2. Standards and Legislation

Medical/surgical masks provide an immediate barrier between the respiratory organs and the surrounding environment. The effectiveness of a face mask or a respirator is determined by two significant factors, the filtration efficiency and fit (facepiece leakage) [28]. Filtration efficiency measures how well the mask filters particles in a specific size range, which includes viruses and other submicron particles, whereas fit measures how well the mask or respirator prevents the leakage around the facepiece. Based on the Food and Drugs Administration (FDA) standards and filtration efficiency, medical masks can be categorised into different categories [29]. These are divided into ASTM level 1, 2 and 3 based on the fluid resistance efficiency [30]. Level 3 gives the highest bacterial filtration efficiency with high resistance to the penetration of body fluids. In Europe, medical masks comply with the requirements of European Standard EN 14683:2019 [31]

However, surgical masks are less effective when compared to respirators. Respirators comprise of tight-fitting protective devices or air purifiers that can prevent very small particles (<5 µm) from passing through a person’s respiratory tract. This is achieved either by removing the contaminants or by providing an independent source of air to breathe. They are named differently in different countries. In the USA, the National Institute for Occupational Safety and Health (NIOSH), determines the filtration efficiency of these respirators and they are classified into N-, R-, and P- series for not oil resistant, somewhat oil-resistant and strongly resistant, respectively [32]. Each of the three series has three different filtration efficiencies at 95, 99 and 99.97%, namely N95, R95, P95, etc. (42 CFR Part 84).

**Figure 3:**
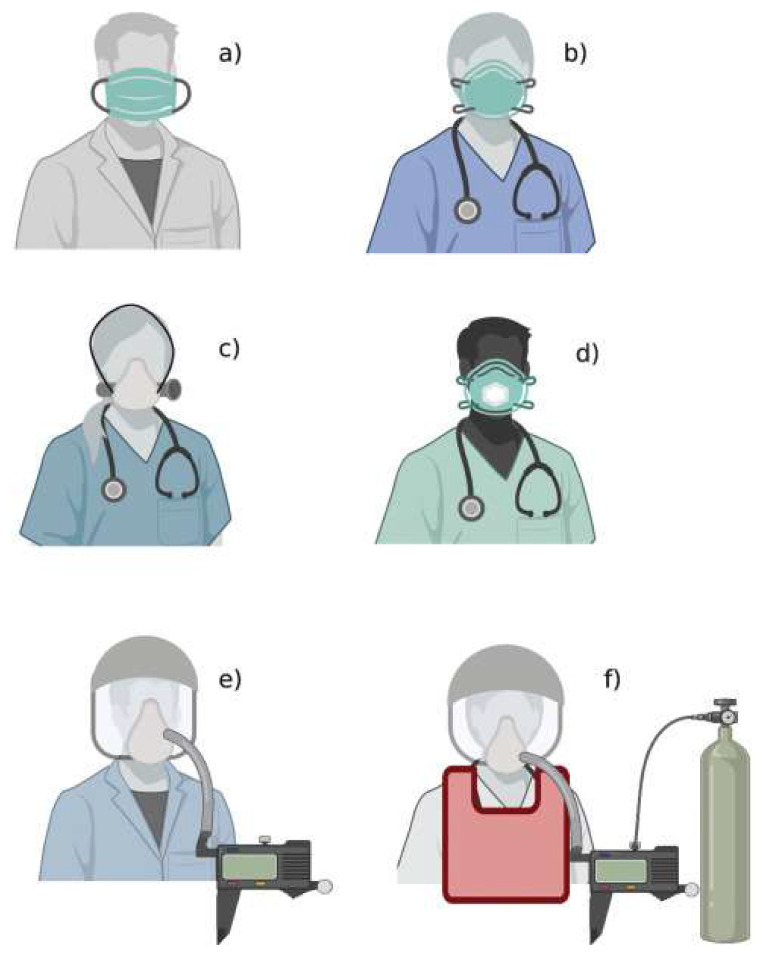
Masks and respirators based on EU standards: (**a**) medical mask; (**b**) filtering facepiece respirator; (**c**) elastomeric respirator; (**d**) filtering facepiece respirator with expiratory valve; (**e**) powered and supplied air respirator; (**f**) atmosphere-supplying respirator. Reused under the terms of the Creative Commons Attribution License, crediting [33], Pulmonology 2020©.

In Europe, the categories of respirators can be classified as filtering half masks (filtering face pieces (FFP)), half masks, powered air-purifying respirator (PAPR) and SAR (atmosphere-supplying respirator [33]. According to European standards, FFPs are further divided into FFP1, FFP2 and FFP3, with an efficiency of 80%, 94% and 99%, respectively (EN 149:2001) [34,35]. The N95 standards are met by FFP2 or 3, which offers the highest filtration efficiency. With the emergence of a pandemic that significantly increased the high demand for face masks, there is a global shortage in the availability of medical face masks. In such a scenario, the Centers for Disease Control and Prevention (CDC) recommends that the public use cloth masks in a public setting to prevent the spread of the virus [36]. This has also led to the development and use of various cloth masks by a large section of society [37]. Figure 3 shows the different categories of respiratory protection equipment (RPE) used by healthcare workers.

NIOSH conducts several precertification tests before legalising the use of a mask or respirator [38]. These include NaCl aerosol challenge, dioctyl phthalate (DOP) test, valve leak and inhalation/exhalation tests to verify the standards (NIOSH Procedure No. RC-APR-STP-0057, 0058 and 0059). In the NaCl aerosol test, the samples are subjected to aerosolised NaCl and the amount of NaCl that passes through the sample determines the filtration efficiency. The DOP test evaluates particle penetration and air flow resistance where the samples are challenged with the particulates of the most penetrating size range (0.3 µm).

**Legislative Guidelines for Selecting and Wearing Facial Protection:**

PPE is only effective when they are used properly and disposed. The World Health Organisation (WHO) has recommended the use of face masks or respirators as a precaution towards preventing the transmission and spread of viruses [39]. In the Republic of Ireland, the Health Safety and Executive (HSE) guidelines and legal responsibilities are followed to reduce the exposure to contaminants to safe levels [40]. The use of RPE is contained within the Control of Substances Hazardous to Health Regulations (COSHH) 2002, risk assessment [41]. The specific requirements for RPE use are:

1. Adequate control of inhalation exposure and to provide effective protection.
2. Be suitable for the intended use.
3. Must be ‘CE’ marked (RPE used must be manufactured according to the PPE Regulations 2002 (PE Directive 89/686/EEC) and the CE marks tell us that the equipment has met the legal requirements for the design [42]).
4. Selected, used and checked regularly by trained people with proper records.
5. Stored and appropriately cleaned.

A basic understanding of the hazardous substances and time of exposure, the form of substances exposed, and the type of work being carried out are required to select the model of RPE required [43].

**Figure 4:**
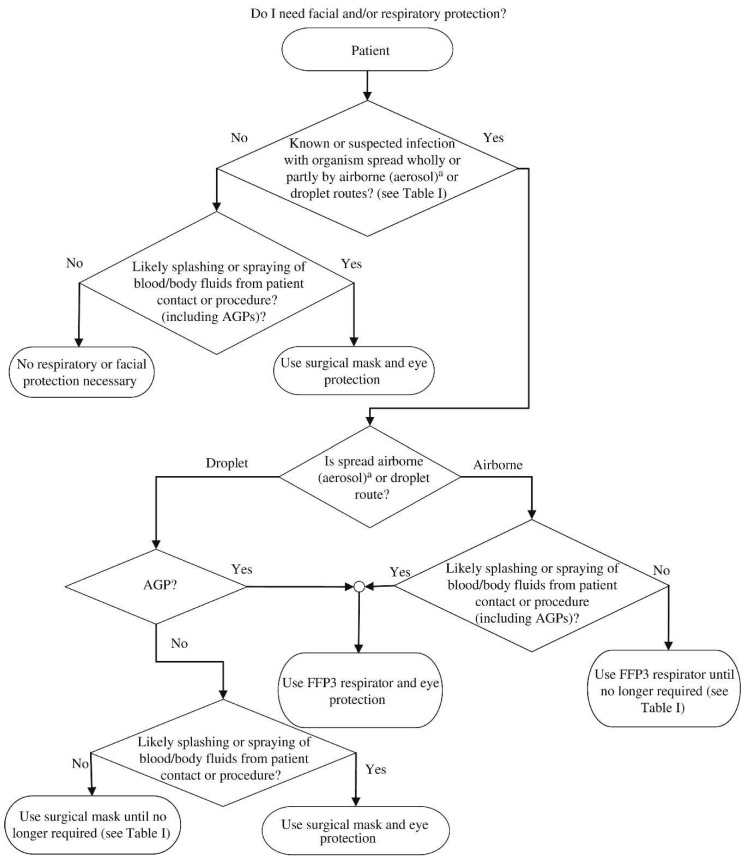
Flow chart illustrating the process to be followed to select the required respiratory protection equipment (RPE). Reproduced with permission from reference [43]. Copyright (2020), Elsevier.

Figure 4 gives the diagrammatic flow chart highlighting the requirement for and selection of appropriate RPE.

However, in any pandemic situation, it should be ensured that any PPE is conserved and prioritised for use by medical and healthcare workers. Considering the same, CDC does not recommend the use of N-95 respirators by the general public even in the case of a pandemic [36,44]. Also, the public needs to follow the proper guidelines as improper use might lead to an increased risk of transmission.

The National Public Health Emergency Team (NPHET) has made several amendments to the Health Protection Surveillance Centre (HPSC) guidance on the use of surgical masks on 22 April 2020, in accordance with the COVID-19 outbreak [45]. These include hand hygiene and maintaining social distancing of 2 m between individuals. Masks should be worn covering the nose and mouth, by holding only elastic tie bands. Once the masks are worn, it is not recommended to move them up and down over the nose and mouth. Furthermore, safe disposal and removal of masks should also be practised [46].

Albeit the fact that FFPR (filtering facepiece respirator) cannot be reused, CDC has currently published guidelines and strategies to manage the spread of pandemics through extensive disinfection and reuse of the FFRs. The FDA issued the Emergency Use Authorisation (EUA) for the decontamination process on 29 March 2020 [47]. However, only the legal manufacturers of FFPR can provide guidance on the strategies that can be applied to decontaminate different models of FFPRs and these should not cause any impact on the performance of the respirator.

## 3. Materials

The membranes used for filtering the submicron particles should also allow the person to breathe and should not clog when the particles adhere to the surface of the masks [48]. The major mechanisms involved in particle removal by fibrous media include gravity settling, inertial impaction, diffusion and electrostatic attraction [49]. It has been reported that particles larger than 0.3 µm are mainly retained by inertial impaction, whereas particles below 0.2 µm are captured by filtration and electrostatic attraction [50].

When there is low airflow through the fibre based filter, smaller particles are filtered at an enhanced ability due to longer time needed to pass through the filter. When the airflow is high, large particle removal is increased due to larger centrifugal forces [49].

The most studied materials in fabricating masks include non-woven fibrous substances such as polypropylene, glass papers, woolen felt. These materials have been used in manufacturing personal protection equipment (PPE) since the beginning of the 20th century. These materials have proven to be capable of withstanding high temperatures during autoclaving without any changes in the structure. Masks are fabricated via melt blowing technique, during which the charges are imparted to the material creating a quasi-permanent electric field providing an adequate filtration of particulate matters (PM) by electrostatic force. The filtration efficiency of the membrane depends upon the structure (pore size, fibre organisation), a charge of the fibres, fibre thickness and diameter, packing density, etc., of the material. It has been concluded that fibres with small diameter and a large surface area that forms small voids when compared to long fibres, leads to increased filtering efficiency.

Several studies have been conducted to theoretically explain the process of filtration through electrostatic attraction and impaction by the fibrous medium [51,52,53,54]. An early study concluded that electret filters (non-woven fibres) hold high filtration efficiency with low air resistance and large dust holding capacity when compared to conventional fibrous filters [55]. The filtration works based on electrostatic forces of attraction between the masks matrix and the aerosol particles and depends on the dielectric property of the material. Hence, polymeric materials with high electrical resistance and stability, such as polypropylene (PP), polyethylene, polyacrylonitrile (PAN), etc., are the best choices for masks and respirators [55]. However, the hydrophilicity of these polymer surfaces is needed to be improved for effective trapping and filtration of aqueous particles [56,57].

**Figure 5:**
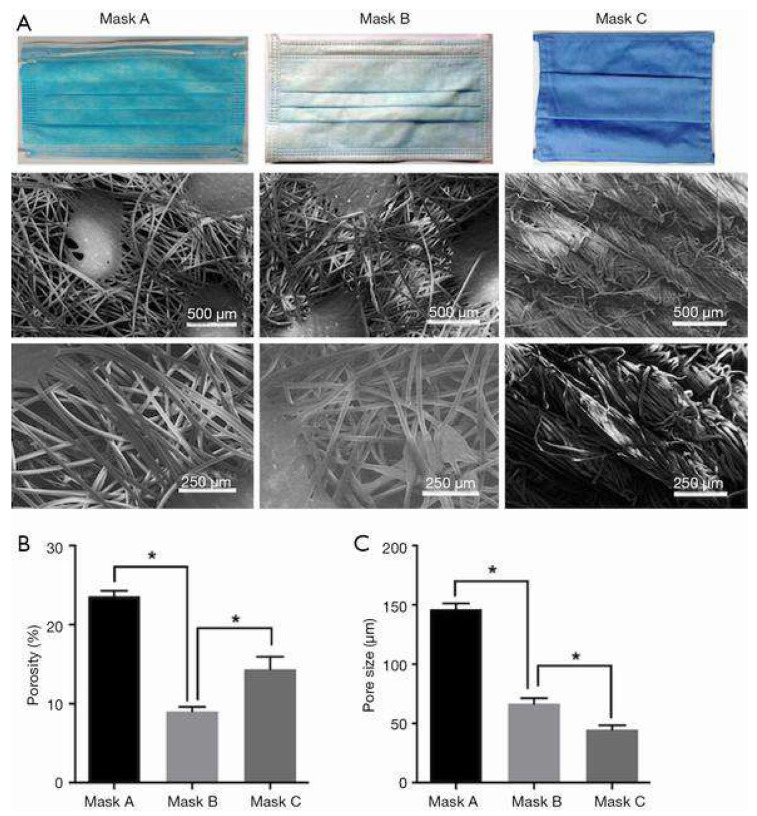
(**A**) SEM images showing the fibre structure of different masks. (**B**) Mask porosity. (**C**) Mask void size distribution. Reproduced with permission from reference [58]. Copyright (2019), Ann Tans Medicine.

There are three major layers in a mask which include i) spun-bond PP fabric, ii) interlayer with melt-blown PP and iii) external layer with spun bond PP fabric similar to layer (i). The middle layer comprises small voids compared to the other two layers and it acts as the filter, stopping the harmful particles from entering the body. A recent study compared three masks with different filtration efficiency, porosity and air flow resistance, Mask A with one filter screen, Mask B with two filters and a washable cloth Mask C [58]. It was concluded that Mask B provided the best filtration owing to its lowest porosity and highest filtering efficiency. Mask A possessed large voids, leading to reduced filtration efficiency whereas Mask C had the highest airflow resistance, leading to breathing difficulties (Figure 5A–C).

**Figure 6:**
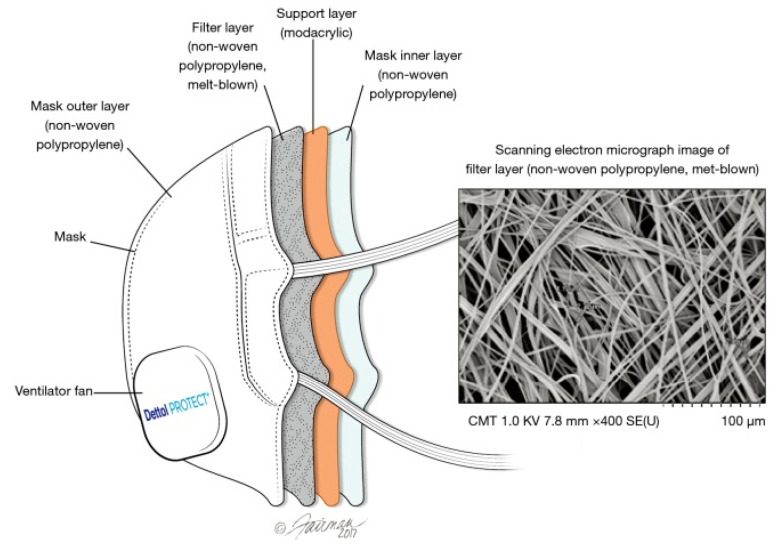
Schematic of different layers of N95 respirator. Reproduced with permission from reference [59]. Copyright (2018), Journal of Thoracic Disease.

A respirator, on the other hand, consists of four different layers of filters [59]. The innermost and outmost layer consists of non-woven PP, which is predominantly hydrophobic, to prevent the moisture from getting absorbed. The middle layers consist of a modacrylic support to provide shape and thickness to the respirator and the non-woven PP layer that captures the unwanted particles. Figure 6 gives the schematic of an N95 mask showing the four layers.

## 4. Mathematical Modelling

The use of mathematical modelling has been significant in broadening the knowledge of transmission mechanisms of infectious diseases while providing theoretical information for the development of public health policy [60,61]. Consider the COVID-19 pandemic; here, the majority of mathematical models used can be generally divided into two specific categories. The first category relates to population-based, Susceptible-Infective-Recovered (SIR)-type models, which are focused on potentially stochastic differential equations [62,63,64], while the second category deals with agent-based models (ABMs) [65,66], in which individuals often communicate on a network structure and transfer infection stochastically. There are certain difficulties posed with the use of both models. Population based models may be too crass to encapsulate specific real-world intricacies [67] while agent-based models are often time-varying, which leads to difficulty in construction along with a loss of accuracy [68]. Many of these models incorporate features of both entities. The correct sequence of dynamical, stochastic, data-driven, and network-based methods will always depend on the question that needs to be addressed [69].

The application of an SIR modelling system was applied to estimate the vitality of face mask use by the general public during an influenza outbreak [70]. Here, estimation of the effects of mask efficacy and coverage (whereby the fraction of population was wearing masks) on basic reproduction number and infection attack rate was used to generate the model. Results show that uncertified masks such as surgical and homemade masks may give an extensive reduction on the reproduction number of the attack rate of the virus. It was also reported that the use of face masks at a population level may delay an influenza pandemic, decrease the attack-rate of the virus and also may reduce transmission to a level where the pandemic may be contained.

Another SIR type model was deployed to determine who should wear masks against airborne infections, i.e., should it be infective or non-infected individuals [71]? Ordinary differential equations and probabilistic cellular automata were used to study how the removal of connections in the random complex network depicting the social interactions among people affects the progression of an infectious disease [71]. The simulation model reveals that if there is a finite number of masks available, then the infective individuals should be those who wear masks [71]. The main challenge here is to correctly identify such individuals and to have accurate corresponding contact tracing procedures in place.

A more complex SEIR model was used to examine the impact of surgical masks on an H1N1 pandemic [72]. Here, the population is divided into two groups: a mask wearing group and a non-mask wearing group. Individuals in each group are characterised by epidemiological status; susceptible (S), exposed (E), infections individuals (I) and recovered (R).

**Figure 7:**
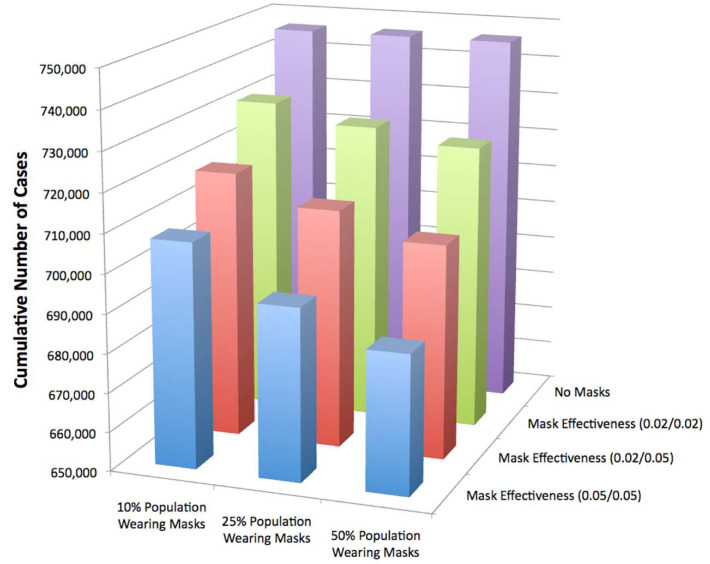
Cumulative number of cases for surgical masks. Reused under the terms of the Creative Commons Attribution License, crediting [72], PLoS 2010©.

As seen in Figure 7, as the mask effectiveness rises, the cumulative number of cases decreases. Also, the number of cumulative cases decreases as the percentage of the population wearing masks increases [72]. Overall, mildly effective masks (20%) could cut the infection number in half. Also, if masks were 50% effective as a source control, the epidemic could be terminated if only 25% of the population wore masks. This being said, another report found that while mask use reduced infection rate, <50% of those in a mask-wearing group reported wearing the mask most of the time [73]. It was found that household uses of masks is associated with low adherence and is limited in controlling seasonal influenza-like illness [73].

Recent studies have focused on the modelling of face masks during the COVID-19 pandemic. One investigation was carried to examine the efficacy of face masks with regard to respiratory virus shedding in exhaled breath [13]. Here, three groups of respiratory viruses were chosen due to their high infection rate (coronavirus, influenza virus and rhinovirus). The model is based on respiratory virus viral shedding of two groups: those wearing face masks and those without. Frequency of detection was studied by using a two-sided Fisher’s exact test and by measuring viral load through the use of an unadjusted Tobit regression model [13]. This model was also used to measure viral shedding without the use of a face mask. Results show that respiratory droplet influenza-transmission may be greatly reduced if a face mask is used. However, this is not the case for aerosol transmission. Results also highlight that the use of face masks by an ill person can greatly reduce the risk of onward transmission [13] which correlates to the study by [71] described above. Within the samples collected without a face mask, the majority of individuals with influenza virus and coronavirus infection did not shed detectible virus in respiratory droplets or aerosols, whereas detectible virus in aerosols was seen in 53% of people with rhinovirus [13]. For those who did shed the virus through respiratory droplets and aerosols, viral load in each tended to be insignificant (after 30 min of exposure). This signifies that extended close contact with an infected individual would be required for transmission to occur [13].

**Figure 8:**
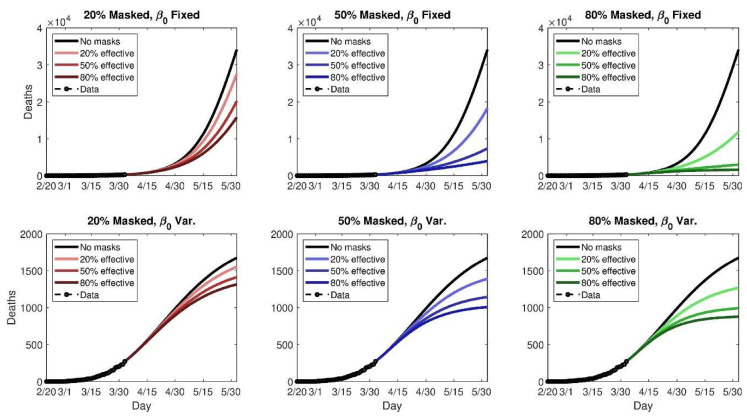
Simulated future (cumulative) death tolls for New York state, using either a fixed (top panels) or variable (bottom panels) transmission rate, β, and nine different permutations of general public mask coverage and effectiveness [66]. Reused under the terms of the Creative Commons Attribution License, crediting [69], KeAi 2020©.

Another COVID-19 based model uses an SEIR system framework to study the potential use of face masks by the general public to curtail the pandemic [69]. A two-group model was adapted here, where the total population is arranged into those who do and do not wear face masks in areas where transmission may occur. This is a deterministic system of nonlinear differential equations [69]. The two main parameters of interest deal with the mask effectiveness as well as population coverage. Simulation analysis based on the mortality rates in New York and Washington state, USA, were conducted. As seen in Figure 8, a snapshot of the simulation shows the future modelled death toll in New York under 18 scenarios.

While these results are hypothetical, the figure suggests that even moderately effective face masks may be beneficial in “flattening” the curve of infection-related deaths. A summary of the data here highlights that 80% adoption of 50% effective masks could prevent 17–45% of projected deaths over a time frame of two months in New York, NY, USA [69]. It was concluded that the use of face masks should be nation-wide as possible and enforced without delay, regardless of the quality of the mask [69].

While the majority of COVID-19 models are focused on population based SIR/SEIR systems [74] have deployed a two model system for predicting the impact of universal mask wearing upon the spread of the SARS-COV-2 virus. The first model type involves a stochastic dynamic network based compartmental SEIR dynamic, which is comparable to previous studies while the second model deploys an individual Agent Based Model (ABM) Monte Carlo simulation [74]. The models used were validated using an empirical data set which includes whether areas have deployed universal masking, daily growth rate and percentage reduction from highest daily growth rates. Results show an almost perfect correlation between early universal masking and successful reduction in daily death rates and daily growth rates [74]. The researchers have emphasised the use of universal masking as a non-pharmaceutical intervention (NPI) to impede the spread of the virus. Universal masking, in combination with social distancing and contact tracing, a “mouth and nose lockdown” may be more feasible than a “full body lockdown” [74]. This is from an economic, social and mental health perspective [74]. Without masking, even with continued social distancing, it is predicted that over half of the population accounted for will be become infected resulting in over a million deaths in a country the size of the UK [74].

In general, mathematical models suggest that face masks (ranging from high to low-grade), with a high adoption rate and high compliance, used in conjunction with other NPIs (e.g., social distancing) will be significant in reducing the spread of an infectious disease and reducing death rates, whether it be related to COVID-19 or similar future epidemics [13,69,70,73,74].

## 5. Recent Advances

Recently, several studies have been performed to improve the efficiency of the respirators and masks against ultra-fine particles such as viruses and other pathogens. These include employing modified filter materials such as nanofibres and nanofibre webs. Also, the virus disinfection capability can be improved by treating the filter surfaces with materials that possess antimicrobial properties. Use of silver nanoparticles (AgNPs) [75], copper oxide [76], iodine [77,78], titanium oxide (TiO_2_) [79], etc, has already been reported in the past decades. With the rapid growth of nanotechnology, fabrication and development of nanomaterials have been improved significantly.

The use of nanofibres in masks and respirators has increased widely since the last decade. Nano-sized fibres offer a very high surface area per unit mass that can improve the capture efficiency as well as other surface areas dependent phenomena such as ion exchange and catalysis [80]. They have small void size, low weight, improved permeability and good interconnectivity of voids [81]. Functionalising the nanofibres with chemicals and nucleating agents also helps in decomposing or deactivating the contaminants, which will reduce the risk of inhaling pathogens and viruses [82]. Electrospinning techniques are most commonly used for the fabrication of nano fibres [83]. Skaria et al. demonstrated that nanofibre filter incorporated surgical masks showed a decrease in air flow resistance and an improved filtration efficiency when compared to commercially available masks [84]. A recent study investigated the mask fit and usability of traditional N95 FFPR with the nanofibre N95 FFPR by analysing before and after nursing procedures [85]. It was concluded that the nanofibre FFR possessed a higher pass rate for the fit testing compared to 3M FFRs. It was also observed to possess a higher bacterial filtration efficiency than the commercially available version in the market. The nanofibre FFPR consisted of partially gelled submicron and nanofibres of PP, and a hydrophilic biocide layer that could effectively inactivate pathogens [86]. It was found that nanofibre FFPR demonstrated significantly better air permeability and higher antibacterial activities than normal N95 respirators and surgical masks.

**Figure 9:**
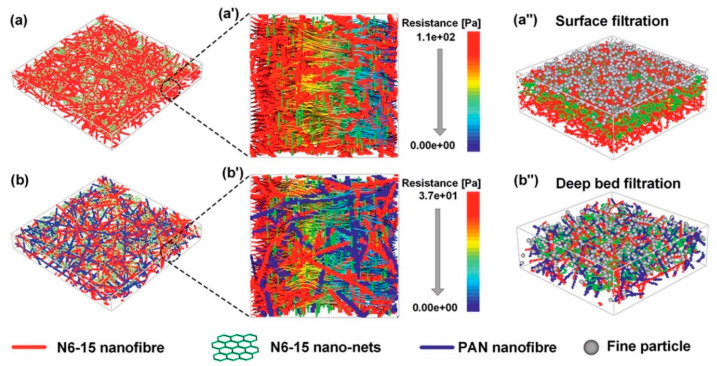
(**a**) 3D N6-15 NFN and (**b**) N6–PAN NNB geometric structures simulated with FibreGeo based on the bulk statistical properties; (**a′**) and (**b′**) are the airflow resistance distributions in the cross-sections of the relevant membranes; (**a″**) surface and (**b″**) deep bed particle loading simulations on 3D tomography images. Reproduced with permission from reference [87]. Copyright (2015), Royal Society of Chemistry.

The introduction of another polymer layer could create excellent filtration media and hence compositing of materials in a filter media has gained much attention. Compositing another layer of filter material with the electret fibres improved the electrostatic charge retention, hence improving the overall filtration efficiency. An ultra-light weight binary structure of nylon 6-polyacrylonitrile nanofibre net (N6-PAN NNB) was fabricated by Wang et al. for the enhanced capture of fine particles with a diameter of 2.5 µm or less (PM 2.5) [87]. N6-PAN NNB was synthesised from nanofibres of polyacrylonitrile (PAN) and polyamide 6-15 (PA6-15) through multi-jet spinning. The composite showed 99.99% filtration efficiency when compared to the commercial fibres available and offered a deep bed filtration pattern in contrast to the surface filtration pattern of normal fibres. A 3D simulation of the structure was also formulated and an air flow resistance model was developed on the basis of the experimental data observed (Figure 9). A very recent study reported the fabrication of a nanofibre composite of PP nanofibres coated with cellulose acetate (CA) and polyvinylidene fluoride (PVDF) to meet the requirements of N95 respirators [88]. Various ratios of CA and PVDF were selected for the study. Sixteen per cent w/v of CA showed the largest first bubble point with a void size of 5.71 µm. The filtration efficiency was solely dependent on the air flow resistance and void faction. Sixteen CA-60 min and 15 CA-30 min samples were observed to meet the requirements of NIOSH for N95 respirators along with a better filtration efficiency. However, two-layer coatings of PVDF were required to meet the standards owing to its small void and thinner fibres.

Further studies were done on incorporating the strength of nanostructures on to the nanofibres to improve the filtration efficiency, reduce pressure drops whilst maintaining the 3D structure. A hierarchically structured fluidised bed filter with agglomerated CNTs was synthesised to filter the aerosol particles [89]. It was found that the manufactured material possessed an enhanced water repellency and high-Quality Factor (QF) when compared to regular filters. Further, a novel electret polyetherimide-silica (PEI-SiO_2_) fibrous membrane was fabricated via an electrospinning technique [90]. A filtration efficiency of 99.992% was obtained with a better self-cleansing property. It was proposed that the permanent dipole orientation of SiO_2_ helps in trapping more charges and presents more stability, providing a chance to adsorb more target particles and pathogens. A recent study demonstrated the fabrication of novel PAN membranes loaded with ZnO, TiO_2_ and AgNPs by electrospinning, and the filtration performances were assessed by NaCl filtration [91]. The TiO_2__F filter displayed the highest filtration efficiency (≈100%), whereas the Ag_F filter showed the highest QF (≈0.06 Pa^−1^). This can be attributed to the smallest diameter and high surface charge of TiO_2__F that improves the particulate attraction and the lower air pressure drop of Ag_F, respectively. The TiO_2__F showed the formation of particle agglomerates owing to its large specific surface area and high interaction between TiO_2_ NPs and PAN fibres. The Ag_F nanofibres also displayed an excellent antibacterial activity towards the gram-negative *Escherichia coli* suspension. Overall, the QF of the fabricated membranes were found to be higher than that of the commercially available nanofibre membranes.

However, the improper disposal and reuse of the masks and respirators might increase the risk of secondary transmissions, especially in the current pandemic situations such as COVID-19. Although several sterilisation methods, such as UV, bleach, ethylene oxide, hydrogen peroxide treatment, have been used to recycle respirators, there had always been drawbacks [92]. Hence, the development of a universal virus decontamination system incorporated in a reusable face mask or respirator to potentially reduce the risk of infection and transmission is a key challenge which is yet to be addressed. Functionalisation of the fibrous filters with materials that possess disinfecting properties or modifying the filter surface with an antigen-specific antibody is the most commonly adopted technique to develop such virus-resistant masks. Metal nanoparticles, such as Cu and Ag, could act as reservoirs for the controlled filtering and will be beneficial for virus trapping [93]. It is demonstrated that the NPs can penetrate through the cell membranes and inhibit the virus attachment. Recent reports suggest that a nanotech surface sanitizer containing TiO_2_ and silver ions was used to disinfect the streets of Milan [94].

Surgical masks modified using a fibrous filtration unit functionalised with sodium chloride (NaCl) were fabricated for virus deactivation [95]. The two major processes involved are i) dissolution of salts by the virus and ii) evaporation induced salt recrystallisation. The virus that is exposed to the supersaturated saline solution becomes inactivated during the drying and recrystallisation phase. This simple virus deactivation strategy could be effective in reducing the risk of transmission in an epidemic or pandemic scenario for PPE’s. An anti-viral material constituting SiO_2_-AgNPs was generated, and was dry coated on a commercial air filter unit. The modified filter was then tested against aerosolised MS2 bacteriophage [96]. The filtration efficiency and the anti-viral efficiency was improved with the increase in the amount of SiO_2_-AgNPs.

**Figure 10:**
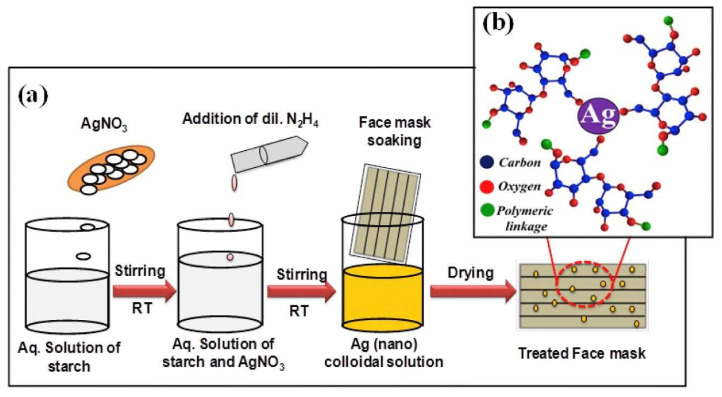
(**a**) Schematic representation of the synthesis of colloidal Ag (nano) solution and its treatment of face mask (**b**) starch capped Ag nanoparticles. Reproduced with permission from reference [99]. Copyright (2018), Elsevier.

Numerous studies have shown the use of silver ions and several silver-based compounds in developing antimicrobial coatings that are known to be highly effective against microorganisms. Although the precise mechanism of deactivation is unknown, most theories state that the positively charged silver ions disrupt the bacterial cell wall and membrane, resulting in an impaired metabolic pathway leading to the death of the cells [97,98]. An antimicrobial coating combining AgNO_3_ and TiO_2_ was applied to respiratory masks and was tested against two bacteria namely *E. coli* and *S. aureus* [75]. The coated masks showed 100% reduction in bacterial growth after 48 h whereas, there was an increase of 25% and 50% of *E. coli* and *S. aureus* counts for the untreated face mask. The nanoparticles did not have any other side effects on the human skin such as inflammation or itching. Hiragond et al. studied the enhanced antibacterial property of commercially available masks by treating it with Ag nanoparticles [99]. Different concentrations of AgNPs were coated onto a surgical mask, taking AgNO3 as the precursor (Figure 10). The best results were obtained for face masks treated with 100 ppm Ag NPs that showed enhanced antibacterial activity of commercial face masks. The silver nanoparticles crossed plasma membranes as well as the lipid bilayer and entered into the cytoplasm, which eventually leads to the destruction of the bacterial cell [80]. This simple method of coating masks will help in reducing the contamination of face masks giving a longer duration of wearability. However, more safety testing regarding the leakage of nanoparticles will be needed for practical applications.

A hybrid PVDF-Ag-Al_2_O_3_ composite was synthesised and used to evaluate the antimicrobial deactivation as well as chemical detoxification [100]. The composite showed high surface-to-volume ratios and excellent antibacterial activity. However, with an increase in Al_2_O_3_ concentration, the Ag NPs were less exposed and hence the zone of inhibition was also reduced. In addition to these, there are several studies on coating herbal extracts to the nanofibres, to develop antimicrobial filtration [101,102]. Recently a PP filter was coated with mangosteen (MG) extract to improve the antibacterial and antituberculosis activities [103]. The MG coated filters showed > 95% bacterial filtration efficiency against three types of pathogens including multidrug-resistant tuberculosis, *Staphylococcus aureus* and *Escherichia coli*.

**Figure 11:**
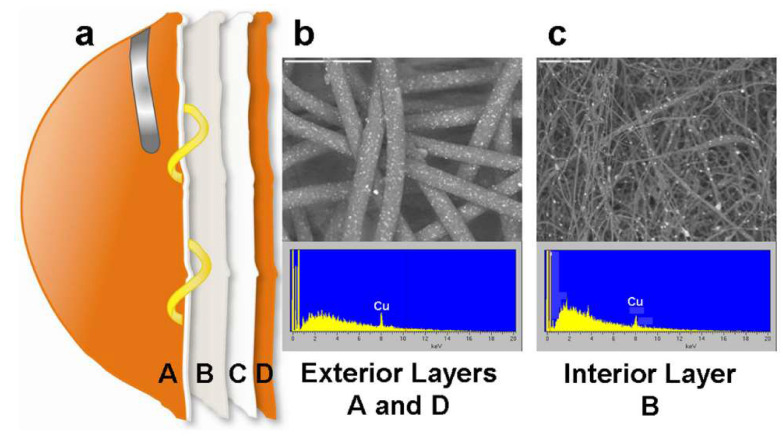
(**a**) Four distinct layers of the respirator, (**b**) SEM and XRD of layer A and D and (**c**) SEM and XRD of layer B. Reused under the terms of the Creative Commons Attribution License, crediting [76], Aerosol and Air Quality Research 2010©.

A novel anti-influenza copper oxide impregnated respiratory face mask was fabricated to protect the wearer from virus droplets [76]. Copper oxide is believed to possess potential anti-viral and antibacterial properties. The filtration efficiency of both control and copper oxide incorporated face masks were found to be the same for both aerosolised viruses of human influenza A virus (H1N1) and avian influenza virus (H9N2) under simulated breathing conditions. The modified face masks showed zero retention of viral titers after 30 min in the case of H1N1, and five-fold times lower than control masks in the case of H9N2 viruses. Figure 11 shows the four different layers of the masks, out of which both layer A, and D, consisted of spunbond polypropylene fabric containing 2.2% weight/weight (w/w) copper particles and the layer B with PP fibres coated with 2% w/w copper oxide particles.

Graphene oxide (GO) has been considered as a promising material for the fabrication of antimicrobial surfaces due to its contact-based antimicrobial activity [104]. A comparative study on the effect of size of GO sheets was conducted using Gram-negative *E. coli* bacteria [105]. The findings concluded that smaller sheet size improved the activity and this was attributed to the high defect density of the smaller sheets and the associated oxidative mechanisms. The anti-viral activity of GO and GO with Ag was investigated against feline coronavirus (FCoV) with an envelope and infectious bursal disease virus (IBDV) without an envelope [106]. GO-Ag was found to inhibit both FCoV and IBDV, whereas GO sheets could only inhibit IBDV. This was ascribed to the synergistic effect of GO sheets and Ag particles contributing to the anti-viral activity. It was concluded that the positively charged lipid membrane was attracted by the GO and this caused the membrane rupture.

**Figure 12:**
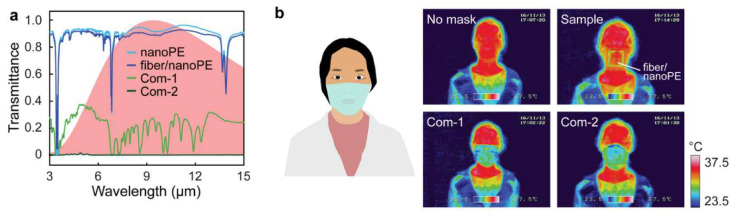
(**a**) FTIR spectra for nanoPE, fibre/nanoPE and two commercially available masks, (**b**) thermal imaging of a bare human face and face covered with the samples. Reproduced with permission from reference [107]. Copyright (2017), American Chemical Society.

Apart from this, other modifications such as thermal stability management, reusability, self-sanitising ability, were also investigated. A novel system of nanofibre/nanoporous polyethylene (fibre/nanoPE), which showed high particulate matter adhesion and capture efficiency was developed [107]. Thermal management aims to enhance/suppress radiative dissipation in a high/low-temperature environment. This material showed an excellent radiative cooling effect owing to the high transmission dispersal of the human body temperature (2.1% for nanoPE, 89.3% for fibre/nanoPE). Incorporating a layer of Ag accommodated the heat loss in low temperatures. The IR transmittance rates were much higher when compared to commercially available masks (Figure 12a). Figure 12b gives the thermal mapping of the human face indicating the temperatures.

Few pieces of research were also conducted on developing self-sanitising and reusable masks, which work based on surface chemistry and roughness. Liu et al. developed a reusable bio-based polyamide-56 nano /nets (PA-56 NF/N) membranes composed of ultra-thin 2D nanonets with synchronised cavity structure [108]. Hydrophobic SiO_2_ nanoparticles were used to demonstrate the reusability and dust-cleaning property of PA-56 NF/N compared to that of H&V HB7613 filters commercially available. Although the filtration efficiency of both materials remained the same, PA-56 NF/N showed relatively less dust holding capacity after mechanical shaking. This was attributed to the cavity structures of nanonets and was consistent with the pressure drop variations during testing.

**Figure 13:**
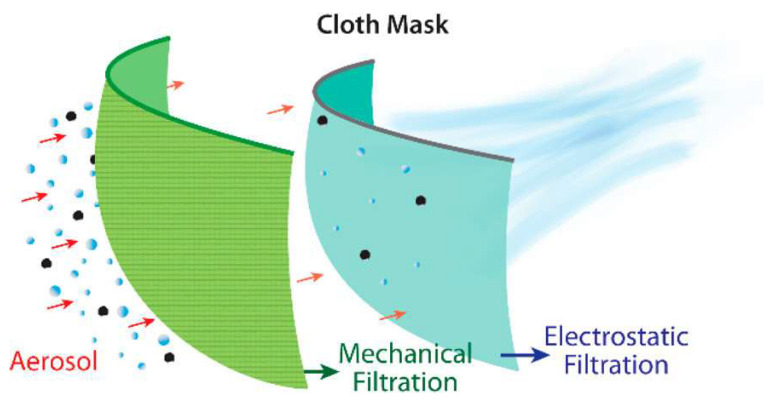
Schematic of the aerosol filtration process of a cloth mask. Reproduced with permission from reference [37]. Copyright (2020), American Chemical Society.

There is still a lot left to be explored in developing high filtration, efficient, reusable, anti-viral and thermally regulated face masks and respirators. Yet, when it comes to unexpected emergence of pandemics such as COVID-19, there would always be a high demand and shortage of surgical masks and respirators. This leads to an increased dependence on cloth-masks despite its low efficiency, especially in low economic countries. Hence, it is quite necessary to evaluate the filtration efficiencies of available fabrics that could provide significant protection from the pathogens. Several reports were already available that assesses the efficacy of cloth masks [109,110]. Recently, Konda et al. studied the aerosol filtration efficiencies (Figure 13) of common fabrics including cotton, silk, chiffon, flannel, several synthetics, and their combinations [37]. NaCl based aerosol testing was used for testing the efficiency of the materials. The differential pressure (ΔP) across the various fabrics was considered as the indicator of comfort and breathability.

It was concluded that cotton, natural silk and chiffon could provide above 50% efficiency, provided they have higher threads per inch with tighter weavings. Cotton quilt with highly tangled fibrous nature provides the best filtration efficiency in the small particle size range. The importance of ‘fit’ was also indicated from the reduction in efficiencies of the fabrics due to leakages around the mask areas.

## 6. Reuse

An examination of the extended use and reuse of respirators found that reuse of respirators could be feasible for non-contact transmission. This was a result of the extended use of masks resulting in comfort issues, which would cause additional touching of the mask increasing infection possibility. Extended use could also allow for degradation of elements such as straps [111]. An examination of five different contamination methods using ultraviolet germicidal irradiation (UVGI), bleach, vaporised hydrogen peroxide, ethylene dioxide micro-wave oven irradiation was carried out by Viscusi et al. [112]. The study primarily focused on the effect of the treatments on the respirators, not the decontaminant effect they had on them. They found that UVGI, vaporised hydrogen peroxide and ethylene dioxide showed potential use for further studies. Treatment using bleach resulted in issues with odour from the mask and the production of low levels of chlorine gas was observed when the masks were hydrated. The microwave oven irradiation resulted in two models of respirators melting in the process and as a result was deemed unfit for use.

When N95 filtering facepiece respirators (FFRs) were treated with ultraviolet germicidal irradiation (UVGI) a greater than three log decrease of influenzas was observed in 80% of facepieces and 46.6% of straps. This method involved treating the equipment with 1 J/cm^2^ of UVGI for 1 min [113]. When UVGI doses of between 120–950 J/cm^2^ were studied, an increase of up to 1.25% in penetration by particles was observed [114]. The main observed effect was on the structure of the mask, materials present saw their strength degrade by up to 90% with considerable variability seen in different brands of respirators. They conclude that the process could be used but would be heavily dependent on the model of the respirator and would have to have a limited number of disinfections for that model.

Examination of the reuse of respirators with COVID-19 using vaporised hydrogen peroxide is proposed by Grossman et al. at Barnes Jewish hospital, Washington [115]. In this process, individuals had their own personal masks that could be disinfected and returned to them. The system incorporated specific collection times of masks and with 200 masks being treated in each cycle. They found that the method could be reproducible within a larger scale to alleviate shortages.

A study on the reuse of three different types of masks, N95, Gauze and Spunlace, that had a most penetrating particle size (MPS) of 118, 461 and 279 nm had penetration rates of 2.6%, 23.2% and 70.0%, respectively [116]. They compared five methods of decontamination using a rice cooker treatment, autoclave, bleach, ethanol and isopropanol. In this study, pressure drop (∆p) and MPS were examined after the decontamination was performed on each type of mask with lower results for both being preferable. The MPS in N95 increased in all treatments with an almost four times increase in size except when using the rice cooker for decontamination. The Gauze saw no real significant change with a decrease seen in rice cooker treatment. The spunlace saw an increase across all the treatments in PMS by roughly 50%. The autoclave had other negative results on the N95 with folding being present in the fabric, this folding could result in changes in the density of the filters and can result in poor effectiveness of the filter. The use of decontamination could potentially be useful for the reuse, but the mask would only have the same protection as gauze with most treatments resulting in an increase in MPS to above 400 nm, which would not be suitable for certain environments.

A further study using rice cookers for decontamination of N95, cloth and surgical mask treated the masks for 13–15 min and compared the results to oven treatment at 100 °C for 15 min [117]. Each mask had been inoculated with 10-µL of 10^6^ colony forming units of MRSA and MS2. The rice cooker treatment obtained over a five-log decrease in MRSA and MR2 in comparison to under a three-log decrease with the oven treatment. This shows potential for the use of steam-based treatment in conjunction with the previous study showing the method can retain structural integrity of the mask whilst also disinfecting it.

## 7. Effectiveness and Fitting

A study on the effectiveness of cotton and surgical masks [118] found that when patients with Covid-19 were instructed to cough five times on a petri dish while wearing a mask, masks saw very little reduction of viral load than without a mask being worn. The viral load in one patient only decreased from 3.53 to 3.26 log copies/mL and 2.27 log copies/mL when a surgical mask and cotton mask were used, respectively. They also found that there was no virus found on the inside of the masks in both cases, but 2.21 and 2.76 log copies/mL were found on the outside of the surgical mask and cotton mask, respectively. In conclusion they found that surgical and cotton masks were ineffective in the prevention of the spread of the virus. This study has received criticism for several aspects; the masks are designed to prevent the spread of the virus when an individual is singing, coughing, speaking, sneezing or breathing. When looking at just coughing it is still recommended that an individual covers their mouth, a further examination would need to be carried out under all the conditions above to find their true effectiveness. It was also noted that having an individual cough in a room first without a mask and then with a mask could lead to contamination of the room. This paper has since been retracted due to a low number of patients being used. A separate study modelling a patient coughing while wearing a face mask found that the mask had a 91% “initial efficiency”, droplets were capable of penetrating the mask and travelling over 1.2 m [119]. They also concluded that if a mask was not worn, droplets would travel at least 70 cm, with the mask the droplets would travel half this distance. This would indicate that the droplets were still capable of penetrating the masks, but their travel distance was limited. Once again, this study examines coughing only and examination of sneezing, breathing, singing and speaking to give a better understanding of their effectiveness. When a surgical mask was examined as an alternative to N95 masks during the SARS pandemic, it was found that surgical masks did not sufficiently filter “submicron-sized” particles [120]. Indicating they would not sufficiently filter the virus due to its size, however, they noted that surgical masks are not designed to protect the wearer from the virus but to protect the individuals around them if they have the virus. As surgical mask void spaces are designed to prevent particles of above 100 µm in diameter, their use for filtering of COVID-19 that can be up to 140 nm in diameter can be seen as negligible [121]. They have been found to be beneficial in reducing coronavirus transmission from “large respiratory droplets and in aerosols” [13]. N95 respirators are capable of filtering particles with a diameter of 100 nm, any virus smaller than that can pass through the filter [122]. Like face masks, respirators will prevent the transference of droplets that are formed from sneezing (≈100 µm) and coughing (≈1 µm).

Respirators are designed to have a tight fit so as the flow of air is coming through the mask and not the sides and each user should have a fit test [123]. Respirators should also undergo a user based seal check before use, this is to ensure that there is no leakage in the respirator [124]. A study examining how many individuals passed the fit test saw that N95 masks (ref1860), N95 masks (re9210) and N100 masks had pass rates of 69%, 55% and 70%, respectively. When the user seal check was examined, 71–75% of them passed, however when this was compared to a Quantitative fit testing, 18–31% of the user seal checks had been incorrectly passed. This would indicate that unless the respirator is fitted and worn correctly it will not give the correct level of protection required. The use of Quantitative fit testing (QNFT) was recommended instead of a user seal check to ensure the mask has a tight fit. Surgical masks were seen to have a very poor fit factor, when five masks were worn together they were only found to have a fit factor of 13.7, the lowest fit factor for a half face respirator is 100 [120]. A separate study of the fit of face masks found that 100% of the masks failed a qualitative fit test on normal breathing. When they were subjected to a QNFT, they obtained fit factors of between 2.5–6.9 when unaided and from 2.8–9.6 when aided [28]. These masks are not designed to be a substitute for a respirator so will obtain low fit factor scores even when multiple masks are used. The fit of these masks is designed to be loose with space between the mask and the face [123].

## 8. Future Perspective and Conclusions

Research communities around the globe are making several efforts to contain and reduce the impact of COVID-19. Unlike the previous epidemics such as SARS and MERS, now the technologies are equipped to control this infection. This growth was seen by the quick actions like identifying the pathogen, rapid development of detection test kits, enhanced research for vaccines or drugs and introducing health care policies in a very short period [125]. However, these steps will not reduce the rapid spread of SARS-CoV-2 virus unless the air borne transmission of the virus is prevented. Use of PPEs is the basic and effective method to use to prevent the transmission. Studies have reported that the virus can remain viable and infectious for hours in aerosols and days depending upon the surfaces [126]. The aerosols formed from coughing or sneezing of an infected patient can spread the virus extensively. The asymptomatic behaviour of infected patients makes it worse to handle this problem. The spreading as well as inhaling of the respiratory droplets, can be drastically reduced by using face masks [37].

Use of innovative and existing technologies to use face masks is one of the primary research interest in this situation. Various research is being done based on the idea of reducing, reusing and recyclability of existing masks, to ease the demand for face masks [127]. New technologies for affordable production methods have been applied, as local ventures can help in the production of masks. Modification of existing materials, as well as an alteration in masks designs, are made to improve the multipurpose functionality in masks. New materials and technologies for mask production or modification of existing surgical masks are reported.

**Figure 14:**
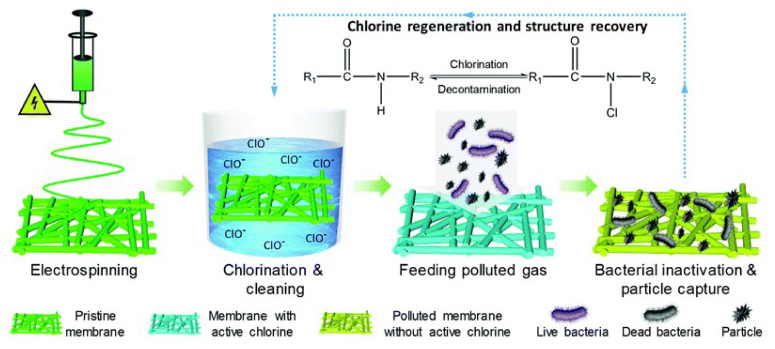
Schematic illustration of the design process of the versatile NFMs with rechargeable biocidal capacity and renewable air filtration performance. Reused under the terms of the Creative Commons Attribution License, crediting [130], Royal Chemical Society 2020©.

Prolonged wearing of N95/N99 masks acts as a cause of contamination as bacteria or viruses are accumulated during the exposure. To solve the problem, Andrey et al. proposed a modification of the N95/N99 mask to provide additional filtration of air and to deactivate the pathogens using low- temperature plasma [128]. Additional to the standard five-layer design of the masks, one plasma layer and additional layer between skin and electrode system were introduced (Figure 14). The low-temperature plasma layer is formed by dielectric barrier discharge (DBD) and its controlled by a switch, which is sensitive to the direction of airflow. Thus, it will discharge during the expiration, and disables it during the inspiration. Whereas, the dielectric layer helps in maintaining the gap and blocking the ultraviolet radiation of discharge for mask wearers. The active oxidising agents formed in plasma (O_3_, H_2_O_2_) that generated positive and negative ions will help in the disinfecting of virus and bacteria. Additionally, the microparticles from the air stream are removed by electrostatic precipitation. These modifications help to increase the service life and the effectiveness of the standard medical masks and to enhance the degree of protection for medical personnel.

Commonly used sterile surgical masks help in reducing via transmission by limiting airborne particles or body fluid droplets from sneezing or coughing. However, in the case of a pandemic situation, it is hard to differentiate the affected patients from others. To tackle this problem, they modified disposable surgical face masks with a temperature sensing material [129]. They patented the modification of surgical masks to detect infectious patients from non-infected using thermochromatic materials. Eisenbrey et al. used temperature-sensitive dyes in masks, which can change colour at 32–33 °C, even for a 1 °C change in temperature. Thus, by the colour change of the thermochromatic material, infected patients can be sorted from the public space. Additionally, adding multiple thermochromic dyes could be used to check the severity of temperature.

Wang et al. successfully synthesised a novel multifunctional material with rechargeable antibacterial efficiency and renewable filtering performance to use in the fabrication of respirators [130]. N-halmine structures were spun on PA nanofibrous membrane (NFM) using the electrospinning technique. The biocidal properties of the NFMs were induced by directly exposing the membrane into free chlorine media (sodium hypochlorite solution). The N–Cl bond formed during exposure will act as an oxidising agent during the contact with the pathogens, leading to the disinfection of materials. This property can be retained again by the exposure of materials to the chlorine solution. Even the 3D structure of the membrane stayed intact after multiple washes. The initial filtration efficiency value of 99.999% was achieved, which is far higher than the required efficiency of N100 face masks (>99.97%). The antibacterial PA-6 NFM could maintain the N100 level even after 10 h. This gives a practical point to use these materials to fabricate face masks or respirators, where rechargeable biocidal property is essential.

Combining the existing practices and research development with industry 4.0 technologies could be the ideal solution to confront and prevent future pandemics [131,132]. In daily life scenarios, messages to encourage wearing face masks in public by asymptomatic individuals have earlier raised resistance. The main reason for this reluctance of the public regarding universal mask-wearing is due to inadequate supply of masks to meet public demand. Another concern is either that people do not wear masks properly, or the possibility that they would become careless in maintaining other infection control measures if they were wearing masks. It is essential to maintain the supply-demand as well as to ensure that the pubic adhere to infection control measures using PPE effectively. Industry 4.0 technologies like Artificial intelligence (AI), 3D printing, Holography, and Virtual reality are being used to tackle this problem.

Industry 4.0 is called the fourth industrial revolution, consisting of advanced manufacturing and information technologies to achieve the customised requirement of different areas of the human being in a shorter time [132]. By using this smart system, essential equipment can be manufactured remotely during the crisis. Designing and development of any medical part are done using advance designing software and digital manufacturing technologies like 3D printing. This provides a smart continuous supply chain of medical disposables and equipment during this crisis [132]. Swenn et al. developed a prototype of a reusable custom made three-dimensionally (3D) printed face mask that can be adopted and used globally. This prototype is created based on individual facial scanning, 3D modelling, and 3D printing [133]. Two components were printed: head fixation band and filter membrane, using polypropylene and polyamide composite as material. These masks are securely fitting around the mouth and nose and are also easy to disinfect for reuse. However, there was dermatological issue consideration of these masks as they made lesions in the nasal bridge after prolonged application.

Artificial Intelligence (AI) is now used to predict the outbreak by making use of geographical mapping and to minimise or prevent the spread of the virus [134]. New AI tools are integrating with surveillance cameras to monitor wearing of face masks and create awareness in public. Individual tracking is implemented to know whether the user had any close contact with a person infected with SARS-CoV-2 in the recent past [135]. Algorithms are modified to recognize the face with a mask to enhance the user-friendliness of smartphones and other technologies. Virtual reality and holograms are being used to create virtual clinics through the application of telemedicine consultations, which will help in reducing public crowding, thus delaying the virus spread. Using these technologies, the community can continuously monitor, collect data, and communicate with each other to develop and manufacture a vaccine, detection kits, healthcare equipment and decide on necessary actions with low human physical participation [136]. The application of this technology will help in fulfilling the requirements of customised face masks, gloves, and collect information for healthcare systems for proper controlling and treating of COVID-19 patients.

The use of face masks will become a vital tool in the future so that humans can move back to a normal day to day setting in dealing with COVID-19. Face mask use is steadily becoming an everyday recommendation from governments with many enforcing their mandatory use outside and the WHO recommending their use. Standards in place prior to the outbreak have enabled the production of quality-controlled equipment that can be used in the current situation and help stem the growth of the pandemic. Current materials used in production including non-woven fibrous substances have been in use since the beginning of the 20th century and have been shown to be still sufficiently viable in their use. Advances in materials have developed coatings such as graphene oxide that are antimicrobial, these can aid both the fabric used and filters helping prevent the transition from the mask to hands and inhalation. Development in nano-sized materials and polymer layers have shown potential in increasing the filtration efficiency of the filters and the use of nanostructures can enhance the structural integrity of the face masks and respirators. The reuse of face masks and respirators has seen limited success, as these are a single use product and extending their use can have negative effects. Ultraviolet treatments were seen to degrade the structure of the mask while chemical based sterilisation methods showed an increase in most penetrating particle sizes in face masks and respirators, reducing their effectiveness. The use of pressurised steam while using steam cookers did however show some success in both the respirators and face mask, with minimal increase in MPS and sufficient sterilisation properties. Development of thermochromatic materials will allow easy identification of individuals showing potential symptoms and the development of reusable materials will greatly help in the coming years. Mathematic modelling of the use of face masks during the pandemic has become a valuable tool, allowing researchers and officials to study the effect of their use over a range of situations. Current legislation, materials and research have provided face mask and respirators that can help deal with the pandemic. However, there is still a significant amount of innovation required for the development of efficient face masks and respirators with anti-viral and thermal regulation properties.
